# RETRACTION: Numb Promotes Autophagy through p53 Pathway in Acute Kidney Injury Induced by Cisplatin

**DOI:** 10.1155/ancp/9803403

**Published:** 2026-04-17

**Authors:** Analytical Cellular Pathology

RETRACTION: Z. Liu, Y. Li, Y. He, J. Wang, “Numb Promotes Autophagy through p53 Pathway in Acute Kidney Injury Induced by Cisplatin,” *Analytical Cellular Pathology*, 2022, 8213683, https://doi.org/10.1155/2022/8213683.

The above article, published online on 27 June 2022 in Wiley Online Library (wileyonlinelibrary.com), has been retracted by agreement between the journal Editor‐in‐Chief, Dimitrios Karamichos; and John Wiley & Sons Ltd. The retraction has been agreed due to concerns with the figures, one of which was also raised on PubPeer [1]. Subsequent investigation identified further concerns. Specifically:

• In figure 1e, there are repeated image elements between the Cisplatin and the NC‐siRNA‐Cisplatin panels on the top row.
• In figure 2, the Numb and p53 bands appear similar when rotated 180 degrees and resized.

After assessment by the Chief Editor, the results and conclusions are deemed unreliable, and therefore the article is retracted.

The authors were unresponsive when informed of the retraction.
